# Supplementary material for: The Systems Biology Research Tool: evolvable open-source software
Source: BMC Syst Biol. 2008 Jun 29;2:55. doi: 10.1186/1752-0509-2-55 (PMC2446383; doi:10.1186/1752-0509-2-55)
Supplement: Additional file 1 — SBRT Archive. An archive of the current version of the Systems Biology Research Tool. [file 1752-0509-2-55-S1.zip › sbrt-1.4.0/doc/developers_guide/api/sbrt/shell/io/package-use.html]

Uses of Package sbrt.shell.io


|  |  |  |
| --- | --- | --- |
| **Overview** \| **Package** \| Class \| **Use** \| **Tree** \| **Deprecated** \| **Index** \| **Help** | |  |
| PREV   NEXT | **FRAMES**    **NO FRAMES**     **All Classes** |  |


---


## **Uses of Package sbrt.shell.io**

| Packages that use sbrt.shell.io | |
| --- | --- |
| **sbrt.shell.io** | Provides classes and interfaces for reading and writing files. |
| **sbrt.shell.io.fba** | Provides classes and interfaces for reading and writing files relevant to Flux Balance Analysis. |
| **sbrt.shell.mng** | Provides classes and interfaces for creating process managers. |
| **sbrt.shell.mng.comb** | Provides classes for creating combinatorics process managers. |
| **sbrt.shell.mng.fba** | Provides classes and interfaces for creating Flux Balance Analysis process managers. |
| **sbrt.shell.mng.geom** | Provides classes and for creating geometry process managers. |
| **sbrt.shell.mng.graph\_theory** | Provides classes and for creating graph theoretical process managers. |
| **sbrt.shell.proc.fba** | Provides classes for converting FBA-related files of different formats. |
| **sbrt.shell.text** | Provides classes and interfaces for defining formats for basic objects. |

Classes in sbrt.shell.io used by sbrt.shell.io

****AbstractMapInputFile****
  
          This abstract class is a skeleton implemenation of text-based
map input files.

****AbstractMapOutputFile****
  
          This abstract class is a skeleton implementation of
`MapTextOutputFile`.

****ComiFile****
  
          This class is used to read column-oriented map input files.

****ComoFile****
  
          This class is used to write maps to text files in a
column-oriented way.

****FileFormat****
  
          This class is used to represent file formats.

****FileLineIterator****
  
          This class is used to iterate through the lines of a file.

****InputFile****
  
          This interface is used to represent input files.

****MapInputFile****
  
          This interface is used to represent input
files that contain maps.

****MapOutputFile****
  
          This interface is used to represent output files that
maps can be written to.

****MapTextFile****
  
          This interface is used to represent text-based
files that contain maps.

****MapTextInputFile****
  
          This interface is used to represent text-based input
files that contain maps.

****MapTextOutputFile****
  
          This interface is used to represent output files that
maps can be written to as text.

****OutputFile****
  
          This interface is used to represent output files.

****RomiFile****
  
          This class is used to read row-oriented map input files.

****RomoFile****
  
          This class is used to write maps to text files in a
row-oriented way.

****SbrtFile****
  
          This interface is used to represent files.

****SerializedFileIterator****
  
          This class is used to iterate over the objects in a serialized
input file.

****SerializedInputFile****
  
          This class is used to read input files containing serialized
objects.

****SerializedOutputFile****
  
          This class is used to write serialized objects to output files.

****SmoFile****
  
          This class is used to write serialized maps to output files.

****TextFileLineIterator****
  
          This class is used to iterate over the lines of an input file.

****TextInputFile****
  
          This class is used to read input files containing text.

****TextOutputFile****
  
          This class is used to represent output files containing
plain text.

Classes in sbrt.shell.io used by sbrt.shell.io.fba

****FileFormat****
  
          This class is used to represent file formats.

****InputFile****
  
          This interface is used to represent input files.

****MapInputFile****
  
          This interface is used to represent input
files that contain maps.

****MapTextFile****
  
          This interface is used to represent text-based
files that contain maps.

****MapTextInputFile****
  
          This interface is used to represent text-based input
files that contain maps.

****OutputFile****
  
          This interface is used to represent output files.

****SbrtFile****
  
          This interface is used to represent files.

****TextInputFile****
  
          This class is used to read input files containing text.

Classes in sbrt.shell.io used by sbrt.shell.mng

****FileFormat****
  
          This class is used to represent file formats.

****SbrtFile****
  
          This interface is used to represent files.

****TextInputFile****
  
          This class is used to read input files containing text.

Classes in sbrt.shell.io used by sbrt.shell.mng.comb

****OutputFile****
  
          This interface is used to represent output files.

Classes in sbrt.shell.io used by sbrt.shell.mng.fba

****ComoFile****
  
          This class is used to write maps to text files in a
column-oriented way.

****FileFormat****
  
          This class is used to represent file formats.

****InputFile****
  
          This interface is used to represent input files.

****MapInputFile****
  
          This interface is used to represent input
files that contain maps.

****MapOutputFile****
  
          This interface is used to represent output files that
maps can be written to.

****MapTextOutputFile****
  
          This interface is used to represent output files that
maps can be written to as text.

****MultiOutputFiles****
  
          This class is used to create multiple output files.

****OutputFile****
  
          This interface is used to represent output files.

****RomoFile****
  
          This class is used to write maps to text files in a
row-oriented way.

****TextInputFile****
  
          This class is used to read input files containing text.

****TextOutputFile****
  
          This class is used to represent output files containing
plain text.

Classes in sbrt.shell.io used by sbrt.shell.mng.geom

****ComoFile****
  
          This class is used to write maps to text files in a
column-oriented way.

Classes in sbrt.shell.io used by sbrt.shell.mng.graph\_theory

****TextOutputFile****
  
          This class is used to represent output files containing
plain text.

Classes in sbrt.shell.io used by sbrt.shell.proc.fba

****OutputFile****
  
          This interface is used to represent output files.

Classes in sbrt.shell.io used by sbrt.shell.text

****FileFormat****
  
          This class is used to represent file formats.

---


|  |  |  |
| --- | --- | --- |
| **Overview** \| **Package** \| Class \| **Use** \| **Tree** \| **Deprecated** \| **Index** \| **Help** | |  |
| PREV   NEXT | **FRAMES**    **NO FRAMES**     **All Classes** |  |


---
